# Association between neonatal hyperbilirubinemia and hypoglycemia in Chinese women with diabetes in pregnancy and influence factors

**DOI:** 10.1038/s41598-022-21114-6

**Published:** 2022-10-10

**Authors:** Jing He, Jiayang Song, Zhijie Zou, Xiaoxiao Fan, Ruixue Tian, Jingqi Xu, Yu Yan, Jinbing Bai, Zhen Chen, Yanqun Liu, Xiaoli Chen

**Affiliations:** 1 Department of gynaecology and obstetrics, Chongqing Health Center for Women and Children (Women and Children’s Hospital of Chongqing Medical University), 120 Longshan Road, Yubei District, Chongqing, 400021 China; 2 Nursing Department, School of Health Sciences, Wuhan University, No. 115, Dong Hu Road, Wuhan, 430071 Hubei China; 3 Emory University Nell Hodgson Woodruff School of Nursing, 1520 Clifton Road, Atlanta, GA 30322 USA

**Keywords:** Diseases, Health care, Risk factors

## Abstract

This retrospective study aimed to investigate the correlation between neonatal hyperbilirubinemia (NHB) and hypoglycemia (NH) in Chinese women with diabetes in pregnancy (DIP), and the influencing factors. All the data were collected July 1, 2017 and June 30, 2020, and 10,558 Chinese women with DIP and live births were included. Two separate multivariate binary stepwise forward logistic regression analysis calculated OR with 95% CI. The prevalence rates of NHB and NH was respectively 3.65% and 5.82% among women with DIP. The comorbidity of both diseases was 0.59%. NH were 1.81 times (OR 1.81, 1.19–2.76) more likely to have hyperbilirubinemia. NHB is positively correlated with NH (OR 1.93, 1.27–2.92). Increased gestational age has a protective effect on both NH (OR 0.76, 0.68–0.85) and NHB (OR 0.80, 0.69–0.92). Abnormal placental morphology is related to NH (OR 1.55, 1.16–2.08) and NHB (OR 1.64, 1.10–2.45). Regarding neonatal outcomes, congenital heart disease (CHD) (OR 2.16, 1.25–3.73; and OR 10.14, 6.47–15.90) was a risk factor for NH and NHB. NHB and NH were significantly correlated in women with DIP. The offspring of DIP with multiple risk factors have a significantly increased risk of neonatal hyperbilirubinemia.

Hyperglycemia during pregnancy is a growing health concern for women and their infants, including pre-gestational diabetes mellitus (PGDM) and gestational diabetes mellitus (GDM)^1^. Diabetes in pregnancy causes short-term and long-term adverse impacts on both pregnant women (e.g., gestational hypertension, abortion, and stillbirth) and neonates (e.g., perinatal death, macrosomia, and infection). Fetuses exposed to maternal hyperglycemia are more likely to have NH and NHB, which significantly threaten the early life of neonates^2,3^. Fetuses with hyperinsulinemia caused by maternal hyperglycemia in the third trimester of pregnancy are more likely to have NH^4^. NH can also be aggravated by maternal hyperglycemia, poor gluconeogenic response, fetal polycythemia, and neonatal perinatal asphyxia^5^. Hypoglycemia usually occurs in the first few hours after birth. Although hypoglycemia is manifested as a short-term symptom, long-term neurodevelopmental sequelae and other adverse effects should be considered^2^.

Fetal hyperinsulinemia drives catabolism via consuming energy, leading to a depletion of oxygen storage, which in turn accelerates fetal growth and increases oxygen demand^6^. An increased erythropoietin concentration is due to chronic fetal hypoxemia, hyperglycemia, and oxidative stress^5^. Increased HbA1c and decreased placental blood flow resulting to decreased placental oxygen supply in pregnant women, further exacerbating the hypoxia problem^7^. The combination of factors contributes to the relative lack of oxygen in the fetus, which is a risk factor for unexplained stillbirth in diabetic pregnancies^8^. Infants of pregnant women with DIP have an increased risk of hyperbilirubinemia compared to normal infants^5^. An increase in red blood cells also leads to an increase in bilirubin. Polycythemia and hyperbilirubinemia are considered as a counter-regulatory mechanism of this relative hypoxic state, which triggers the secretion of erythropoietin (EPO) and increases the production of red blood cells^7,9,10^. It also leads to an increased incidence of hypoxia, such as respiratory distress syndrome and hypoxic-ischemic encephalopathy^11^.

As the fetus is in the uterus with hyperglycemia, the physiological and pathological mechanisms of NH and NHB may be correlated, which has rarely been studied. Research evidence shows that there is no correlation was found between NH and NHB in pregnant women with GDM, which may be related to the small sample size^12^. Based on the physiological mechanism, this study attempted to increase the sample size and collect more comprehensive variables in Chinese diabetic pregnant women to understand the relationship between NH and NHB. We hypothesized an association between NH and NHB in DIP, and further analyzed the influencing factors.

## Results

The results of baseline maternal characteristics of Chinese women with DIP were shown in Table 1.The mean age, height, pregestational BMI, gestational age at delivery, gravidae, and parity was 31.24 ($\pm$ 4.36) years, 158.53 ($\pm$ 5.02) cm, 22.37 ($\pm$ 3.17) kg/m^2^, 38.36 ($\pm$ 1.52) weeks, 2.49 (± 1.56) times and 0.48 ($\pm$ 0.58) times, respectively. The incidence of NH among Chinese women with DIP was 5.82% and NHB was 3.65%, with a 0.59% combined incidence of both.

**Table 1:** Characteristics of participants and delivery information (n = 7816).

Characteristic	Classification	No. (%)
Age, years	18–24	323 (4.13)
	25–29	2661 (35.05)
	30–34	3112 (39.82)
	35–39	1402 (17.94)
	40–44	294 (3.76)
	≥ 45	24 (0.31)
Height, cm	< 155	1401 (17.92)
	155–159	2950 (37.74)
	160–164	2447 (31.31)
	165–169	862 (11.03)
	≥ 170	137 (1.75)
	Unknown	19 (0.24)
Pregestational BMI, kg/m^2^	< 18.5	612 (7.83)
	18.5–24.9	5572 (71.29)
	25.0–29.9	1298 (16.61)
	≥ 30.0	155 (1.98)
	Unknown	179 (2.29)
Gestational age at delivery, weeks	37–37^+6^	806 (10.31)
	38^+6^	2524 (32.29)
	39^+6^	2813 (35.99)
	> 40	1668 (21.34)
	Unknown	5 (0.06)
Gravidae	1	2588 (33.11)
	2	2057 (26.32)
	3	1446 (18.50)
	4	919 (11.76)
	≥ 5	797 (10.20)
	Unknown	9 (0.12)
Parity	0	4394 (56.21)
	1	3122 (39.94)
	2	273 (3.49)
	3	17 (0.22)
	≥ 4	1 (0.01)
	Unknown	9 (0.12)
Delivery mode	Spontaneous delivery	3555 (45.48)
	Cesarean operation	4261 (54.52)

Univariate analysis of maternal demographic information showed that pregnant women who were older, more gravidae, heavier pre-pregnancy weight, and larger pre-pregnancy BMI were more likely to have NH. In clinical variables, the risk of NH can also be aggravated by insulin use, pre-eclampsia, higher OGTT 1-h glucose, higher OGTT 2-h glucose, and smaller gestational age. Among the obstetric factors, cesarean section, pregnancy with scar uterus, placenta previa, threatened premature labor (TPTL), fetal distress, abnormal placental morphology, and postpartum hemorrhage were more likely to lead to NH. In neonatal outcomes, the birth length, Apgar 1 min ≤ 7, Apgar 10 min ≤ 7, gender (female), macrosomia, neonatal weight, head circumference, asphyxia, neonatal respiratory distress syndrome (NRDS), pneumonia, hypoxic ischemic encephalopathy (HIE), congenital heart disease, anemia, and hyperbilirubinemia were more likely to develop hypoglycemia in neonates. Data with statistical differences were shown in Table 2.

**Table 2:** Risk factors for neonatal hypoglycemia and hyperbilirubinemia (n = 7816).

	NH (n = 455)	Non-NH (n = 7631)	*P *value	NHB (n = 285)	Non-NHB (n = 7531)	*P *value
**Maternal and obstetric factors**						
Age, years	31.80 $\pm$ 4.40	31.16 $\pm$ 4.33	.002	30.71 $\pm$ 4.12	31.22 $\pm$ 4.34	.052
Gestational weeks	38.47 $\pm$ 0.94	38.71 $\pm$ 0.94	< .001	38.50 $\pm$ 1.11	38.70 $\pm$ 0.93	.003
Gravida	2.67 $\pm$ 1.55	2.46 $\pm$ 1.55	.006	2.28 $\pm$ 1.45	2.48 $\pm$ 1.53	.027
Parity	0.47 $\pm$ 0.53	0.48 $\pm$ 0.58	.831	0.31 $\pm$ 0.54	0.48 $\pm$ 0.58	< .001
Pre-pregnancy weight, kg	57.36 $\pm$ 9.45	56.19 $\pm$ 8.44	.005	5 $7.17\pm$ 10.34	56.22 $\pm$ 8.42	.132
Pre-pregnancy BMI, kg/m^2^	22.31 $\pm$ 4.65	21.82 $\pm$ 4.55	.026	22.11 $\pm$ 4.98	21.84 $\pm$ 4.54	.318
Insulin use	58 (12.75)	552 (7.50)	< .001†	25 (8.77)	585 (7.77)	.535†
Gestational hypertension	39 (8.57)	490 (6.66)	.115†	28 (9.82)	501 (6.65)	.036†
Pre-eclampsia	27 (5.93)	256 (3.48)	.006†	13 (4.56)	270 (3.59)	.386†
OGTT fasting glucose	4.86 $\pm$ 0.70	4.85 $\pm$ 0.62	.733	4.94 $\pm$ 0.74	4.84 $\pm$ 0.62	.013
OGTT 1-h glucose	10.33 $\pm$ 1.73	10.00 $\pm$ 1.64	< .001	10.32 $\pm$ 1.87	10.0 $1 \pm$ 1.63	.002
OGTT 2-h glucose	8.82 $\pm$ 1.69	8.5 $2 \pm$ 1.58	< .001	8.81 $\pm$ 1.93	8.52 $\pm$ 1.57	.004
Cesarean section	339 (74.51)	3922 (53.28)	< .001†	162(56.84)	4099 (54.43)	.422†
PROM	103 (22.64)	1813 (24.63)	.338†	102 (35.79)	1814 (24.09)	< .001†
Scar uterus	140 (30.77)	1610 (21.87)	< .001†	44 (15.44)	1706 (22.65)	.004†
Placenta previa	22 (4.84)	168 (2.28)	.001†	5 (1.75)	185 (2.46)	.450
TPTL	3(0.66)	81 (1.10)	.376†	8 (2.81)	76 (1.01)	.004†
Fetal distress	72 (15.82)	739 (10.04)	< .001†	51 (17.89)	760 (10.09)	< .001†
APM	66 (14.51)	627 (8.52)	< .001†	41 (14.39)	652 (8.66)	.001†
Postpartum hemorrhage	463.38 $\pm$ 261.20	427.02 $\pm$ 189.72	< .001	461.18 $\pm$ 316.75	427.94 $\pm$ 188.61	.005
**Neonatal outcome**						
Gender (female)	231(50.76)	3454(46.92)	.011*	120(42.11)	3565(47.34)	.082†
Weight, g	3409.10 $\pm$ 470.10	3342.20 $\pm$ 399.23	.003	3281.65 $\pm$ 543.23	3348.53 $\pm$ 397.60	.041
Head circumference, cm	34.91 $\pm$ 1.10	34.66 $\pm$ 1.01	< .001	34.43 $\pm$ 1.43	34.68 $\pm$ 1.00	.004
Length, cm	50.00 $\pm$ 1.68	49.86 $\pm$ 1.58	.059	49.48 $\pm$ 2.19	49.88 $\pm$ 1.56	.003
Apgar 1 min	3 (0.66)	10 (0.14)	.008	1 (2.11)	7 (0.09)	< .001
Apgar 10 min	1 (0.22)	2 (0.03)	.042	1 (0.35)	2 (0.03)	.006
FGR	6 (1.32)	80 (1.09)	.645†	16 (5.61)	70 (0.93)	< .001†
Macrosomia	49 (10.77)	378 (5.14)	< .001†	18 (6.32)	409 (5.43)	.519†
Neonatal asphyxia	12 (2.64)	37 (0.50)	< .001†	24 (8.42)	25 (0.33)	< .001†
NRDS	2 (0.44)	6 (0.08)	.020†	3 (1.05)	5 (0.07)	< .001†
Neonatal septicemia	9 (1.98)	41 (0.56)	< .001†	26 (9.12)	24 (0.32)	< .001†
Neonatal pneumonia	29 (6.37)	102 (1.39)	< .001†	60(21.05)	71 (0.94)	< .001†
HIE	7 (1.54)	8 (0.11)	< .001†	5 (1.75)	10(0.13)	< .001†
CHD	35 (7.69)	141 (1.92)	< .001†	74 (25.96)	102(1.35)	< .001†
Neonatal anemia	5(1.10)	15 (0.20)	< .001†	9 (3.16)	11 (0.15)	< .001†
Hyperbilirubinemia	46 (7.69)	239 (3.25)	< .001†			
Hypoglycemia				46 (16.14)	409 (5.43)	< .001†

Values, mean $\pm$ SD or n (%).

*NH* neonatal hypoglycaemia, *NHB* neonatal hyperbilirubinemia, *GWG* gestational weight gain, *BMI* body mass index, *ICP* intrahepatic cholestasis of pregnancy, *OGTT* oral glucose tolerance test, *PROM* premature rupture of membranes, *TPTL* threatened premature labor, *FGR* fetal growth restriction, NRDS, neonatal respiratory distress syndrome, *HIE* hypoxic ischemic encephalopathy, *CHD* congenital heart disease, *APM* abnormal placental morphology.

^†^Chi-squared.

In the maternal factor, height, parity, age > 35 years, family history of diabetes, BMI more than 30 kg/m^2^, weight gain during pregnancy, and OGTT fasting glucose had no significant effect on NH. Other complications during pregnancy (ICP, thalassemia in pregnancy, hysteromyoma, anemia in pregnancy, hypothyroidism during pregnancy, viral hepatitis type B, thrombocytopenia in pregnancy, Group B Streptococcus (GBS) infection, gestational hypertension, chorioamnionitis had no significant effect on neonatal hypoglycemia. Among the obstetric factors and neonatal outcome, premature rupture of membranes (PROM), Apgar 5 min ≤ 7, oligohydramnios, polyhydramnios, placental abruption, umbilical cord around neck, and fetal growth restriction had no significant difference in the influence of NH (Table S1).

**Figure 1:**
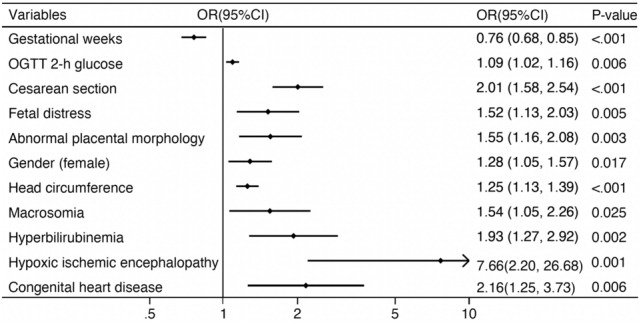
Maternal factors and neonatal outcomes associated with neonatal hypoglycemia.

Larger gestational weeks (OR 0.76, 95% CI 0.68–0.85), elevated OGTT 2-h glucose (OR 1.09, 95% CI 1.02–1.16), cesarean section (OR 2.01, 95% CI 1.58–2.54), fetal distress (OR 1.52, 95% CI 1.13–2.03), abnormal placental morphology (OR 1.55, 95% CI 1.16–2.08), neonatal gender(female) (OR 1.28, 95% CI 1.05–1.57), head circumference (OR 1.25, 95% CI 1.13–1.39), hypoxic ischemic encephalopathy (OR 7.66, 95% CI 2.20–26.68), congenital heart disease (OR 2.16, 95% CI 1.25–3.73), macrosomia (OR 1.54, 95% CI 1.05–2.26), and hyperbilirubinemia (OR 1.93, 95% 1.27–2.92) were statistically significant and associated with NH in multivariate analysis. These factors increased the risk of NH (Fig. 1).

Univariate analysis of the independent variables that we included, parity, were associated with NHB. In addition, having gestational hypertension, higher OGTT fasting glucose, higher OGTT 1-h glucose, and higher OGTT 2-h glucose were more likely to develop NHB. We conducted continuous variable analysis for gestational age, and there were differences in the incidence of NHB between both (*P* < 0.05). NHB is at higher risk through premature rupture of membranes, scar uterus, threatened premature labor, fetal distress, abnormal placental morphology, chorioamnionitis, and postpartum hemorrhage effects.

In neonatal outcomes, the lower neonatal weight, shorter birth length, Apgar 1 min ≤ 7, and Apgar 10 min ≤ 7 had an impact on the development of NHB. Neonates with fetal growth restriction, asphyxia, neonatal respiratory distress, septicemia, pneumonia, hypoxic ischemic encephalopathy, congenital heart disease, anemia, or hypoglycemia were more likely to develop NHB. All significant data results were presented in Tables 2 and S1.

**Figure 2:**
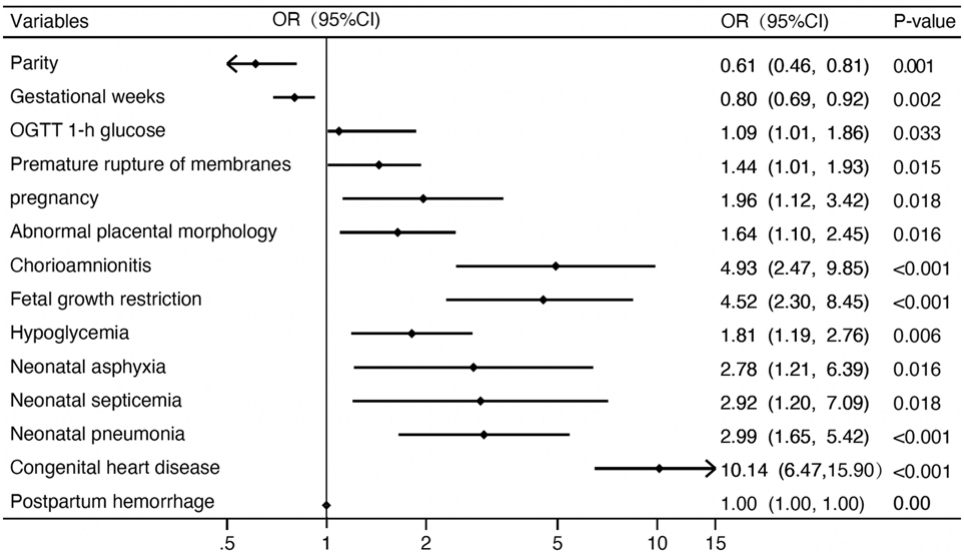
Maternal factors and neonatal outcomes associated with neonatal hyperbilirubinemia.

A new risk assessment regression model for NHB was established. Our results showed that OGTT 1-h glucose (OR 1.09, 95% CI 1.01–1.86), pregnancy with thalassemia (OR 1.96, 95% CI 1.12–3.42), abnormal placental morphology (OR 1.64, 95% CI 1.10–2.45), chorioamnionitis (OR 4.93, 95% CI 2.47–9.85), fetal growth restriction (OR 4.52, 95% CI 2.30–8.45), neonatal pneumonia (OR 2.99, 95% CI 1.65–5.42), congenital heart disease (OR 10.14, 95% CI 6.47–15.90), asphyxia (OR 2.78, 95% CI 1.21–6.39), septicemia (OR 2.92, 95% CI 1.20–7.09), and hypoglycemia (OR 1.81, 95% CI 1.19–2.76) increased the risk of NHB. Larger gestational weeks (OR 0.80, 95% CI 0.69–0.92) and increased parities (OR 0.61, 95% CI 0.46–0.81) decreased the incidence of bilirubinemia in the newborn (Fig. 2).

## Discussion

Our results showed that the incidence of hyperbilirubinemia in neonates with hypoglycemia is 1.81 times higher than that in neonatal non-hypoglycemia, and that NH is an important independent risk factor for NHB. Moreover, increased gestational age has a protective effect on NH and NHB, but abnormal placental morphology is related to the risk factors. In neonatal outcomes, congenital heart disease was a risk factor for NH and NHB, respectively.

Furthermore, neonates with hyperbilirubinemia were 1.93 times more likely to have hypoglycemia than those without hyperbilirubinemia. NH mainly occurs in the first few hours after birth, but this result illustrates the correlation between NHB and the physiological and pathological mechanism of hypoglycemia^5^. Theoretically, fetal hyperinsulinemia caused by maternal hyperglycemia is the core of NH and increased oxygen demand^13^. At the same time, the influence of hyperglycemia on the placenta blood glucose increases neonatal hypoxia^7,14^.

The relationship between NH and NHB may be mutually influencing, and we conducted two regression analyses of its possible influencing factors. In our model, OGTT 2-h glucose had an effect on NH. Abnormal postprandial glucose levels suggested impaired glucose tolerance and β cell dysfunction on a physiological basis^15^. Maternal postprandial hyperglycemia affects the fetus through the placenta, leading to fetal and neonatal hyperinsulinemia, which was one of the main causes of NH^16^. Therefore, it is necessary to manage the blood glucose during pregnancy for neonatal outcomes.

The rate of cesarean section, macrosomia, and fetal distress in Chinese women with DIP was increased by obstetric screening techniques. Neonates delivered by cesarean section are more likely to develop hypoglycemia^17^. Our study showed that cesarean section was an independent risk factor for NH, and the probability of hypoglycemia was 2.01 times that of neonates delivered by non-cesarean section. Adverse pregnancy outcomes of DIP during pregnancy include fetal overgrowth, representing a high incidence of neonatal hypoglycemia and macrosomia^18^. Our study also found that macrosomia and larger head circumference increased the risk of neonatal hypoglycemia. Neonatal macrosomia and large head circumference increase the difficulty of vaginal delivery, which is also the reason for the high rate of cesarean section.

In our study, morphology and fetal distress were risk factors for NH. Abnormal placental morphology is prone to intrauterine growth restriction and fetal distress. While the probability of hypoglycemia increased in the fetuses with diabetic mothers due to glucose stimulation of placental blood vessels, to date there is little research on the correlation of abnormal placental morphology and DIP and further exploration is needed. In the hypoglycemia regression model, we also found a positive correlation between neonatal gender, with male neonates being 1.28 times more likely to have hypoglycemia than female neonates.

In NHB, there was a negative correlation for gestational age, which is consistent with previous studies^19^. Previous studies have shown that multiparity are more prone to abnormal blood glucose of pregnant women due to age and other factors^20,21^. In our study, there was a negative correlation between parity and NHB, the mechanism of which is unknown. The reason that parity is a protective factor for neonatal hyperbilirubinemia may be due to less use of oxytocin and fetal head aspirators in the second fetus, thereby reducing complications^22^. In mothers with DIP and thalassemia, there is a higher prevalence of anemia during pregnancy and hyperbilirubinemia in neonates^23^. The prolonged half-life of red blood cells during iron deficiency anemia leads to an incorrect increase in HbA1c levels, which may exacerbate the adverse pregnancy outcomes associated with blood glucose^24^.

In addition, we observed an increased risk of NHB in maternal chorioamnionitis, neonatal asphyxia, and septicemia. These factors may be related to fetal infection and hypoxia when the mother has chorioamnionitis. At the same time, fetal infection and hypoxia trigger erythrocytosis and destruction, increasing the risk of neonatal asphyxia and septicemia^25,26^. Moreover, hyperglycemic stimulation of DIP leads to decreased placental function, vasculopathy, and exacerbation of infection, which increases the risk of fetal hypoxia and neonatal hyperbilirubinemia^27^.

In the physiological and pathological mechanisms, NHB may be a sign of potential intrauterine hypoxia, and the occurrence of neonatal sepsis, ischemia, hypoxia, and anemia is significantly positively correlated with NH^28^. The lack of detection of neonatal erythrocytes as a direct predictor of NHB in our study only showed a possible correlation with adverse neonatal outcomes in the regression model. Previously studies have reported that neonates of mothers with diabetes have a higher risk of cardiovascular system abnormalities^6^, and we also observed a positive correlation between congenital heart disease and NH. Neonatal anemia was positively associated with both NH and NHB. It is necessary to strengthen the nursing of neonatal anemia, timely find the risk of adverse outcomes, and give corresponding countermeasures. These findings highlight the correlation between NH and NHB, suggesting that there are physiological and pathological links.

As far as we know from the search results of the systematic review related to the content of this study, this study is a population-based observational study with a large sample size and relatively comprehensive inclusion of relevant variables. We analyzed the effect of NH on NHB in diabetic mothers, providing evidence for home management of neonatal jaundice. The effects of maternal factors, obstetric factors and neonatal outcomes on NH and NHB respectively, as well as the relationship between the both, were also analyzed.

The limitations of this study should also be considered. First, some of the data of NHB were obtained from EMR in neonatal unit after birth or from obstetrics unit transferred to the neonatal unit. The rest of them were obtained through telephone follow-up, which may have recall bias. Second, obstetricians have some differences in the combination of OGTT results and other examination results of pregnant women who have not found abnormal blood glucose before pregnancy to diagnose GDM or pre-pregnancy diabetes. Third, blood glucose control during pregnancy has an important impact on the outcome of delivery, but we failed to obtain this data. Fourth, pre-pregnancy weight is self-reported, and there may be measurement tool and recall bias.

In conclusion, the blood glucose level of DIP is very important for the outcome of childbirth. These findings highlighted the existence of an association between NH and NHB with common risk factors, suggesting physiological and pathological associations between them. Therefore, it is necessary to strengthen the close monitoring of jaundice in neonates with postnatal hypoglycemia to reduce the adverse effects of high bilirubin on the growth and development of neonates.

## Materials and methods

### Study period and participants

**Figure 3:**
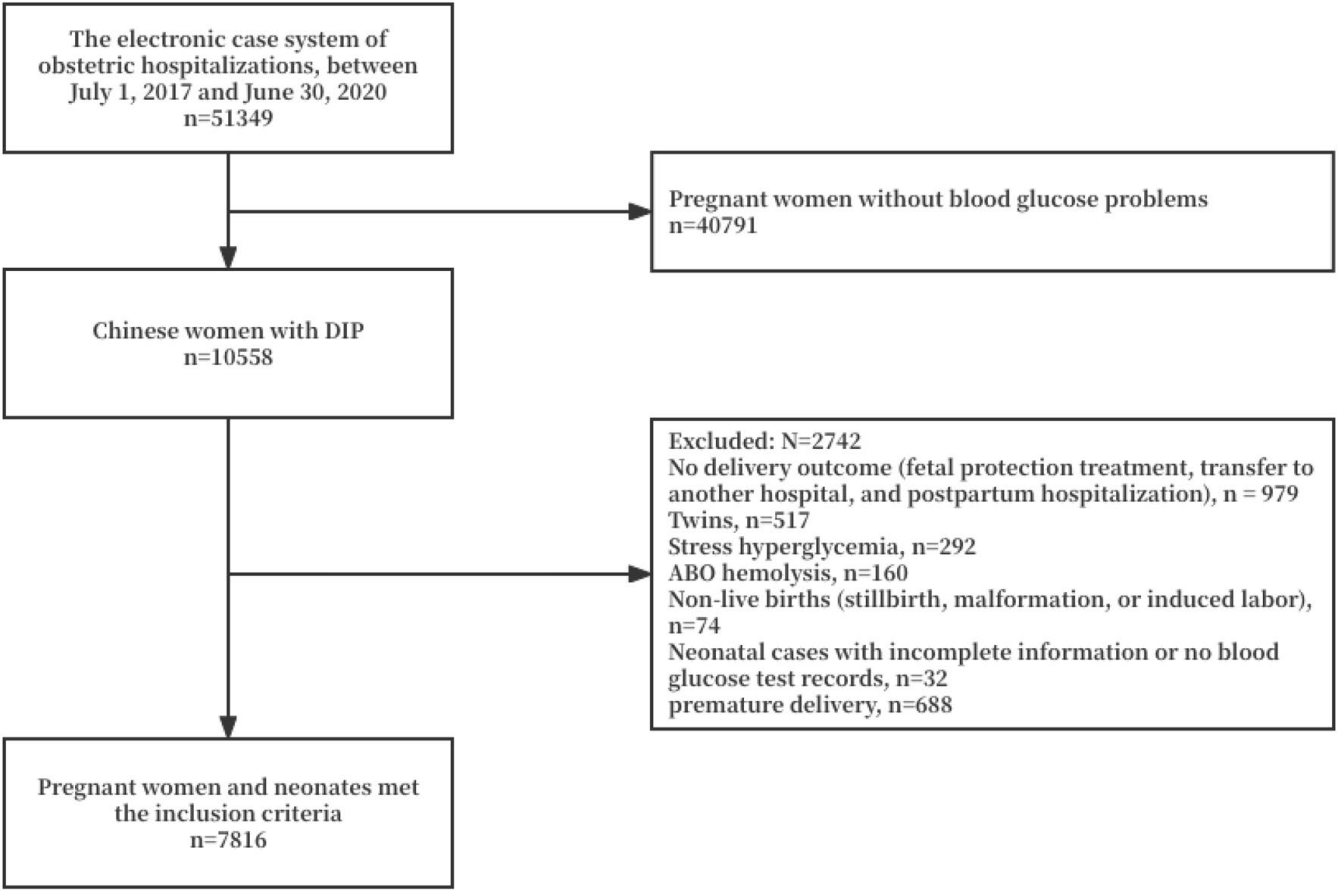
Flow chart for study population.

This study retrospectively analyzed 10,558 Chinese women with DIP and the neonatal outcomes by reviewing electronic medical records (EMR) between July 1, 2017 and June 30, 2020 from the Chongqing Health Center for Women and Children in Chongqing, China. Written informed consent from the patients was not required due to the retrospective nature of the study and the study was conducted in accordance with the national legislation and institutional requirements. This study was approved by the ethics committee of the Chongqing Health Center for Women and Children (Number: 2020–022), all procedures were performed in accordance with the ethical standards. This study has passed the registration of Chinese Clinical Trial Registry (ChiCTR) with the registration label ChiCTR2000040588. In addition, this study was conducted in accordance with the strengthening the reporting of observational studies in epidemiology (STROBE) statemen^29^. Infants with a clinical diagnosis of neonatal hemolytic jaundice, congenital malformation, stillbirth, or neonatal death were excluded. In this study, 8,504 cases of pregnant women and neonates met the inclusion criteria (Fig. 3).

## Diagnosis and definitions

Pregnant women who had been diagnosed with diabetes at the obstetric visit, had a fasting plasma glucose (FPG) during pregnancy ≥ 7.1 mmol, or whose blood glucose after 75-g oral glucose tolerance test (OGTT) (or random blood glucose) ≥ 11.1 mmol were diagnosed with PGDM^30^. Diagnostic criteria for the 75-g OGTT were that the three blood glucose levels should be respectively lower than 5.1, 10.0, and 8.5 mmol/L before, 1-h, and 2-h after glucose administration^30^.

The most commonly used clinical threshold for treatment was plasma glucose (P-glucose) below 2.6 mmol/L^31^. Retest blood glucose > 2.6 mmol/L was given routine care, but again, less than 2.6 mmol/L was transferred to neonatology department^31^. The blood glucose monitoring of neonates was conducted using the GK Dual test instrument produced by On·Call Company in China. Newborns in routine obstetric care were monitored by blood sampling at the end of the heel, and newborns transferred to the neonatal intensive care unit (NICU) were monitored by venous blood sampling. Hyperbilirubinemia was diagnosed when percutaneous bilirubin exceeded the 95th percentile of the Bhurani nomogram monitoring time point during bilirubin monitoring or phototherapy neonates. Hyperbilirubinemia was acquired within the first seven days of life.

In maternal demographic data, pre-pregnancy body mass index (BMI) is calculated as weight recalled from the first obstetric examination. Gestational weight gain (GWG) is calculated as the difference between maternal weight at delivery and pre-pregnancy weight (kg). Scar uterus is defined as having a history of cesarean delivery. Gestational hypertension is defined as the first appearance during pregnancy of systolic blood pressure ≥ 140 mmHg and/or diastolic blood pressure ≥ 90 mmHg measured on at least two occasions in the same arm. Pre-eclampsia is an increase in blood pressure and proteinuria that occurs after 20 weeks of gestation. Hypothyroidism is present before pregnancy or detected at the first obstetric examination. Intrahepatic cholestasis of pregnancy (ICP) is diagnosed in women with elevated serum total bile acid (TBA ≥ 10 μmol/L), the main symptom is pruritus, and other causes of liver dysfunction were excluded^32^. Polyhydramnios refers to amniotic fluid volume (AFV) ≥ 8 cm or amniotic fluid index (AFI) ≥ 25 cm. Oligohydramnios refers to AFV ≤ 2 cm or AFI ≤ 5 cm.

In obstetric data, abnormal placental morphology included wheel placenta, sail placenta, racket placenta, and accessory placenta. Postpartum hemorrhage is defined as blood loss ≥ 500 ml within 24-h after delivery. Fetal growth restriction (FGR) is defined as a fetus whose birth weight was two standard deviations below the average weight for the same gestational age, or below the 10th percentile of normal weight for the same age. A macrosomia is a newborn with a birth weight greater than 4000 g.

## Statistical analysis

Data were collected through double entry (JH and JS) into Excel database. Data was imported and analyzed using SPSS, version 23. Descriptive information was generated, the frequency and percentage of categorical variables and the mean and standard deviation or median (IQR) of continuous variables were calculated. If the variables were continuous (normally or non-normally distributed), an unpaired Student *t* test or Mann–Whitney *U* test was used to compare the differences between the groups. *Chi-squared* tests were used to compare percentages. The missing data of all variables were within 10% without any addressed.

Chinese women with DIP were divided into groups based on neonatal hypoglycemia, non-hypoglycemia, neonatal hyperbilirubinemia, and non-hyperbilirubinemia. For univariate variables, Student *t* test and *Chi-squared* tests were used to compare differences, and *P* < 0.05 was considered statistically significant. Variables with univariate differences were used for multivariate binary stepwise forward logistic regression analysis to produce OR and 95%CI, and parameters of statistical significance (*P* < 0.05) were determined. We compared ignoring missing values within 10% with multiple interpolation methods to evaluate the impact of missing values. The Apgar score was a continuous variable, but there were very few newborns with abnormalities. We converted the Apgar score to ≤ 7 or > 7 for analysis.

## Supplementary Information

Supplementary Information.

## Data Availability

Data sets generated during the current study have been shared in the research manager (ResMan) repository, (http://www.medresman.org.cn/login.aspx). The datasets generated during the current study are not publicly available due to the research data is still being analyzed, but are available from the corresponding author on reasonable request.
