# Supplementary material for: Association between neonatal hyperbilirubinemia and hypoglycemia in Chinese women with diabetes in pregnancy and influence factors
Source: Sci Rep. 2022 Oct 10;12:16975. doi: 10.1038/s41598-022-21114-6 (PMC9550859; doi:10.1038/s41598-022-21114-6)
Supplement: Supplementary file 1 — Supplementary Information. [file 41598_2022_21114_MOESM1_ESM.pdf]

**Association between neonatal hyperbilirubinemia and hypoglycemia in Chinese women with diabetes in pregnancy and influence factors**

Jing He,[1,2] Jiayang Song,[1] Zhijie Zou, [1]Xiaoxiao Fan,[1] Ruixue Tian,[1] Jingqi Xu,[1] Yu Yan,[2] Jinbing Bai[3], Zhen Chen,[2*] Yanqun Liu,[1*] Xiaoli Chen[1*]

[1]Nursing Department, School of Health Sciences, Wuhan University, No.115, Dong Hu Road, Wuhan 430071, Hubei, China
[2]Obstetrics department, Chongqing Health Center for Women and Children, Yubei District 120 Longshan Road, 400021, Chongqing, China
[3]Emory University Nell Hodgson Woodruff School of Nursing, 1520 Clifton Road, Atlanta, GA 30322, USA
* Correspondence:
Zhen Chen, Yanqun Liu, and Xiaoli Chen are co-corresponding authors.
E-mail address and telephone numbers:
zhenzhenwyg@126.com (Zhen Chen), +86 023 60354515;
liuyanqun1984@163.com (Yanqun Liu), +86 027 68759710;
chenxl201313@163.com (Xiaoli Chen), +86 027 68759710.

ORCID: 0000-0001-7654-1872 (Jing He)

Table S1.Risk factors and outcomes for neonatal hypoglycemia and hyperbilirubinemia (n=7816)

| | NH (n=455) or (mean ± SD) | Non-NH (n=7361) or (mean ± SD) | P-value | NHB (n=285) or (mean ± SD) | Non-NHB (n=7531) or (mean ± SD) | P-value |
|---|---|---|---|---|---|---|
| Maternal and obstetric factors | | | | | | |
| Height, cm | 158.73 ± 5.09 | 158.5 ± 5.12 | .478 | 158.70 ± 5.32 | 158.56 ± 5.01 | .646 |
| Age > 35 years | 111 (24.40) | 1609 (21.86) | .205† | 52 (18.25) | 1668 (22.15) | .118† |
| Diabetes family history | 49 (10.77) | 806 (10.95) | .898† | 28 (9.82) | 827 (10.98) | .548† |
| Thalassemia | 12 (2.64) | 278 (3.78) | .212† | 17(5.96) | 273 (3.63) | .040† |
| Hysteromyoma | 26 (5.71) | 345 (4.69) | .317† | 18 (6.32) | 353 (4.69) | .204† |
| Anemia in pregnancy | 81 (17.80) | 1536 (20.87) | .117† | 52 (18.25) | 1676 (8.98) | .300† |
| Hypothyroidism | 46 (10.11) | 655 (8.90) | .380† | 25 (8.77) | 676 (8.98) | .906† |
| Viral hepatitis type B | 27 (5.93) | 433 (5.88) | .964† | 15 (5.26) | 445 (5.91) | .649† |
| Thrombocytopenia | 18 (3.96) | 204 (2.77) | .140† | 6 (2.11) | 216 (2.87) | .447† |
| GBS infection | 16 (3.52) | 307 (4.17) | .496† | 19 (6.67) | 304(4.04) | .029† |
| GWG, kg | 12.62 ± 5.60 | 12.21 ± 4.70 | .083 | 12.23 ± 5.13 | 12.24 ± 4.75 | .983 |
| BMI > 30 kg/m2 | 14 (3.08) | 141 (1.92) | .085† | 9(3.16) | 146 (1.94) | .147† |
| ICP | 10 (2.20) | 214 (2.91) | .379† | 10 (3.51) | 214 (2.84) | .508† |
| Placental abruption | 6 (1.32) | 54 (0.73) | .165† | 0 (0.00) | 60 (0.80) | .130† |
| Obstetric factors | | | | | | |
| Oligohydramnios | 55 (12.09) | 761 (10.34) | .236† | 29 (10.18) | 787 (10.45) | .882† |
| Polyhydramnios | 6 (1.32) | 60 (0.82) | .255† | 4(1.40) | 62 (0.82) | .293† |
| Chorioamnionitis | 5 (1.10) | 83(1.13) | .955† | 7 (2.46) | 81 (1.08) | .030† |
| UCAN | 113 (24.84) | 2060 (27.99) | .146† | 84(29.47) | 2089 (27.74) | .521† |
| Apgar 5min | 1 (0.22) | 4 (0.05) | .176† | 5 (0.35) | 4 (0.05) | .051† |

Values are (mean ± SD) or n (%), † Chi-squared. NH, neonatal hypoglycemia; NHB, neonatal hyperbilirubinemia; UCAN, Umbilical cord around neck; GBS, group B streptococci; CP, intrahepatic cholestasis of pregnancy; GWG, gestational weight gain.
